# Recent applications of parylene and pyrolyzed parylene in sensors and devices: a review

**DOI:** 10.1007/s00216-025-06150-1

**Published:** 2025-10-17

**Authors:** He Zhao, B. Jill Venton

**Affiliations:** Department of Chemistry, University of Virginia, Charlottesville, USA

**Keywords:** Parylene, Microfluidic, Immunoassay, Pyrolyzed parylene, Neurotransmitter, LIG

## Abstract

Parylene is an insulating polymer that is coated in thin layers on sensors and devices. It exhibits excellent gas and moisture impermeability, enabling it to function as a biocompatible encapsulation layer. Parylene is commonly deposited by chemical vapor deposition (CVD) on substrates, with coating thicknesses ranging from nanoscale to microscale, depending on the amount of precursor loaded into the parylene coater. Parylene was deposited on cell culture devices made from 3D printing resins, and it prevents leachates that are toxic to cells, enabling long-term cultures. Pristine parylene is also used for sealing implantable sensors, the cantilever of atomic force microscopy (AFM), and microelectromechanical systems (MEMS), preventing damage in harsh environments. In addition to the intrinsic insulating properties of parylene, parylene is pyrolyzed at high temperatures, inducing structural reformation and converting it to a conductive material. Pyrolyzed parylene is utilized for electrochemical sensing of molecules, coupled with cyclic voltammetry (CV), fast scan cyclic voltammetry (FSCV), or immunoassays. For example, pyrolyzed parylene sensors can be used for in vivo tracking of neurotransmitters. A CO_2_ infrared laser can be an alternative method to induce the graphitization of parylene films. Laser-induced graphene (LIG) is used to fabricate microsupercapacitors. A plasma deposition method for parylene has recently been developed, enabling the creation of a crosslinked structure that favors the immobilization of antigens for immunoassay fabrication. Overall, parylene is easy to coat on a variety of materials and provides biocompatible insulation of analytical devices, with easy pyrolysis to carbon used in sensors.

**Graphical abstract:**

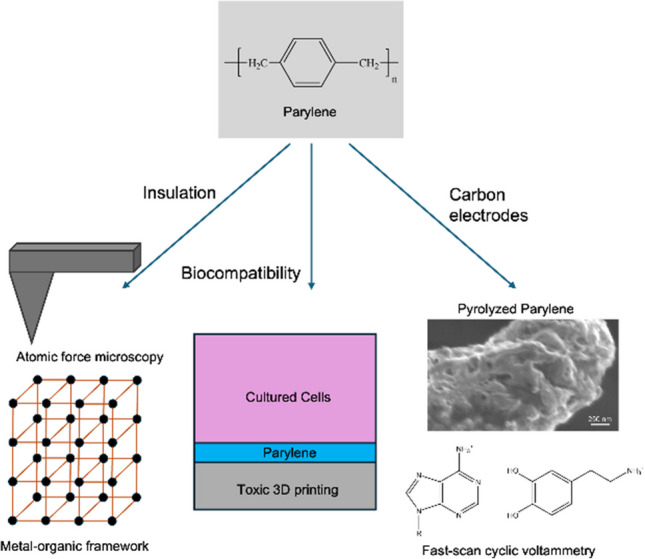

## Introduction

Polymer coatings are often used in analytical sensors and devices as insulators and as substrates for carbonization. Polyimide (PI) is a polymer that has been used for years as an electrical and thermal insulator, as it possesses good temperature resistance, dielectric properties, high mechanical strength, and chemical stability. However, polyimide is primarily spin-coated onto substrates, which limits the substrates that can be used [1–3]. Poly(p-xylylene), also known as parylene, is a benzene-rich polymer that can be chemically vapor-deposited on various substrates. There are many derivatives of parylene, N, C, D, VT4, and AF4, that denote different functionalizations [4–6]. The chemical vapor deposition (CVD) ensures that parylene is uniformly coated in a thin layer on the sample surface, allowing for better coating of non-flat substrates, such as devices and wires [7–9]. Parylene has demonstrated excellent performance in insulating various systems due to its biocompatibility, which surpasses that of polyimide. Many microelectronics and medical devices are coated with parylene, especially C and N derivatives, to prevent corrosion and leaching, as it is chemically inert, flexible, and transparent [10–12]. Parylene N, whose molecular weight is 208.3 g/mol, exhibits excellent dielectric properties and can cover complex surfaces due to its high crevice penetration [13, 14]. The chlorine atom in parylene C, whose molecular weight is 277.2 g/mol, enables it to possess better resistance to gases, moisture, and chemicals than parylene N, but correspondingly lowers the crevice penetration performance [15]. Therefore, parylene derivatives can be customized to achieve different purposes. This review covers applications of parylene for analytical devices and sensors, highlighting its advantages to provide biocompatible thin layer insulation and carbon electrodes in a controlled fashion.

## Methods to fabricate parylene analytical devices

### Parylene deposition

Parylene is chemically vapor-deposited on different substrates, typically in dedicated parylene coaters [25, 26]. The precursor, di-para-xylene, is loaded in the vaporizer, which raises the temperature to 150 °C to vaporize the dimer under vacuum. The dimers are broken down into reactive monomers after undergoing pyrolysis at a high temperature, 650 °C. The monomers are transferred to the deposition chamber at room temperature, where they deposit as poly(para-xylene), parylene [5, 14, 27]. By adjusting the amount of dimer powder loaded, coating thicknesses are varied from nanoscale to microscale.

Considering the complex geometries of target substrates, the advantage of CVD of parylene is that the substrate surface can be wholly and uniformly coated with parylene due to vapor deposition [28, 29]. Thus, cylindrical, spherical, or complex-shaped devices can be fully coated. This cannot be achieved by spin coating, which is commonly utilized for the deposition of polyimide, but it only coats the top of flat surfaces [30, 31]. Due to its hydrophobic property, parylene is coated on various devices to prevent water incursion [10, 32]. Because the deposition chamber is maintained at room temperature, materials sensitive to high temperatures can be coated with parylene.

Conventional parylene deposition is based on the thermal deposition of monomers at room temperature, resulting in a chain-linked structure [5, 14, 27]. An alternative method to parylene coat substrates is plasma deposition, which generates reactive radicals during the pyrolysis of parylene dimer and achieves a 100–200 nm coating within 20 min [33, 34]. Under high plasma, parylene C chains are highly crosslinked, which prevents the generation of small fragments. Plasma-deposited parylene C exhibits a more amorphous crystal structure, which alters its physical and chemical properties, decreasing the sp^2^/sp^3^ratio and surface roughness [20]. The plasma-assisted deposition method achieves a smoother surface than the conventional thermal method. Annealing at high temperatures induces shrinkage in parylene deposited by both methods, but with different ratios: 90% and 50% decreases for thermal- and plasma-deposited parylene, respectively [19, 35]. The higher shrinkage ratio of thermally deposited parylene is caused by its higher proportion of structural crystallinity. Raman spectra revealed that C-H in-plane deformation and CH_2_wagging and twisting vibrations, present in thermally deposited parylene, are absent in plasma-deposited parylene [36, 37]. Thus, the different structures might be useful for different applications in devices or sensors.

## Applications of parylene as an insulator for devices

### Parylene C protects resin-3D-Printed devices

**Fig. 1:**
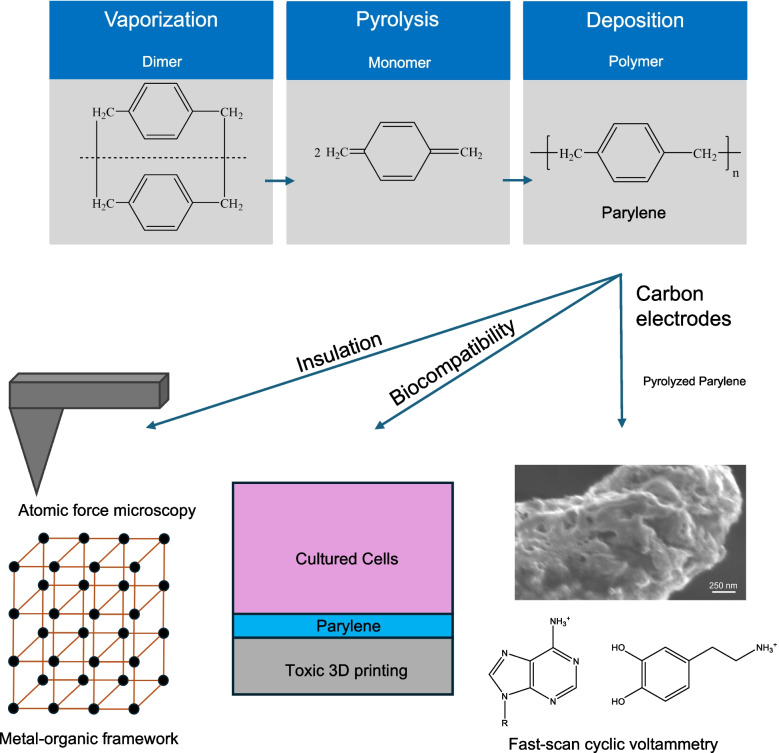
Overview of parylene application on various techniques

Microfluidic devices used in conjunction with cell or tissue culture must be biocompatible and not leach materials into the device or allow materials from the device to enter the microfluidic structure. A new method for quickly fabricating microfluidic devices utilizes resin 3D printing. However, the instability and high cytotoxicity of most photopolymerizable resins render them unsuitable for making organ-on-chip and cell culture devices, particularly for sensitive cells [38–40]. Additionally, small molecules can be absorbed by the resin, which can easily degrade over time (Fig. 1). Furthermore, the temperature used for tissue studies, 37 °C, accelerates the erosion rate [41].The Pompano group discovered that most cultivated cells die in untreated resin devices but survive after coating the resin surface with parylene C [42]. The parylene layer prevents moisture from penetrating through the resin structure and cultured cells from being affected by the resin surface, especially its toxicity [43]. The impermeable characteristic of parylene also enables the resin to resist delamination over time, providing indirect contact between target cells and 3D-printed devices [12, 44, 45]. Therefore, parylene C–treated resin 3D printing can be used for cell culture for multiple cycles. The Pompano group tested three resins: Asiga PlasClear, FormLabs Clear, and MiiCraft Clear, and parylene coating made all the devices biocompatible. Thus, parylene C coating effectively blocks the cytotoxicity of these resins for sensitive primary cells. Parylene coating of microfluidic platforms was further applied to a 3D-printed multiple-compartment organ-on-chip (MOOC) platform [46].

The chemical inertness of parylene enables coated devices to resist damage from aggressive solvents, acids, and bases. However, the adhesion of parylene to the surface of the structure is a limitation that restricts its use in systems requiring immersion in liquid environments. Anisotropic potassium hydroxide (KOH) wet etching was applied to parylene films, which were separately deposited on silicon, silicon dioxide, and silicon nitride wafers, to test the stability. 3-Methacryloxypropyltrimethoxy silane, known as Silquest A-174 silane, functions as an adhesion-promoting silane to form covalent bonds between the hydroxyl groups of silicon-based surfaces and paraxylene radicals [15, 47].

### Parylene coatings improve scanning electrochemical and atomic force microscopy tips

Many physical and biological processes are investigated using scanning probe microscopes to measure micro-/nanoscopic processes [48–52]. The measurement point where the tip is placed is typically determined by the application of a feedback loop. The feedback loop uses the Faradaic current, tunneling current, or ion current, respectively, in scanning electrochemical microscopy (SECM), scanning tunneling microscopy (STM), and scanning ion conductance microscopy (SICM) [51, 53, 54]. These techniques can be combined with atomic force microscopy (AFM) to more accurately locate the target point and prevent damage caused by the sample surface colliding with the tip during preparation [55–62]. One challenge in combining SECM and AFM is that the entire cantilever generates a large charging current, which disturbs the Faradaic responses generated when the tip interacts with the sample surface. Insulating materials, including electrophoretic paint, silicon nitride, silicon oxide, polyfluoroethane, and photoresist, have been utilized by different groups to insulate the cantilever [63–68].

**Fig. 2:**
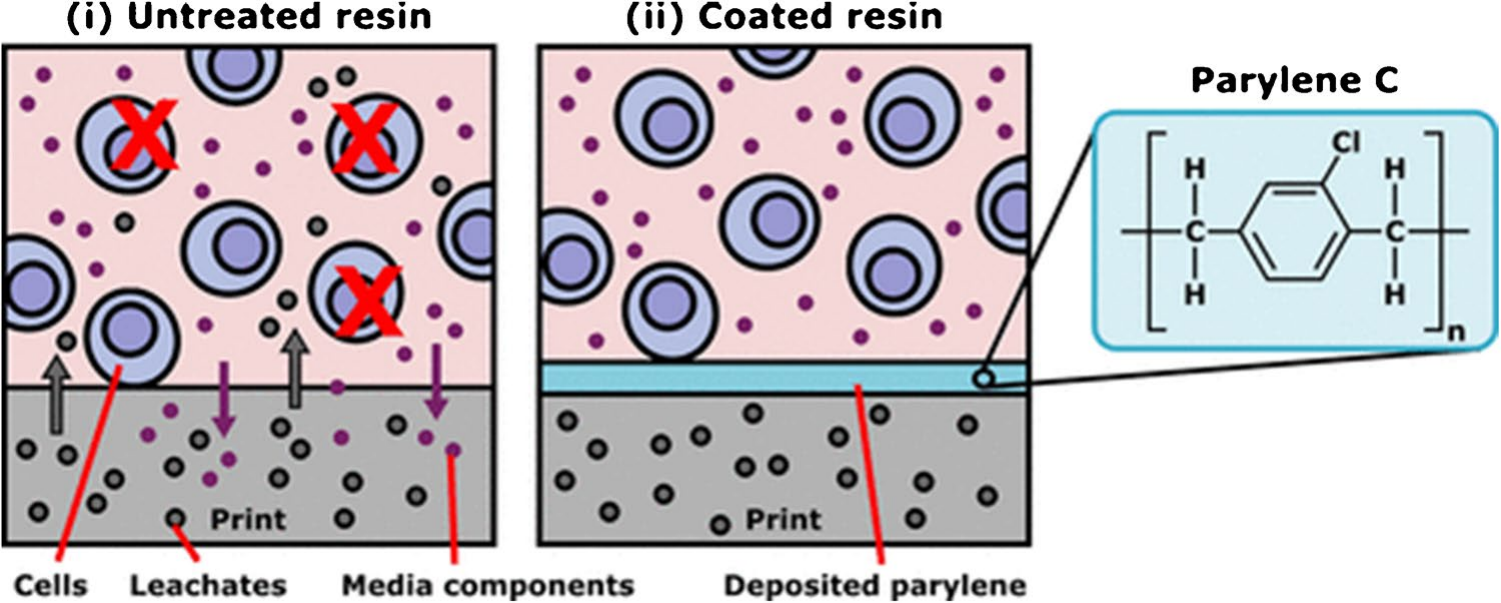
Principle of protection by parylene C coating. (**i**) Toxic leachates (gray-filled circles) diffuse out of 3D-printed materials and interact with cells in the culture while causing a loss of material stability. Meanwhile, the absorption of small molecules (purple dots) into the prints may deplete media components or contribute to material degradation. (**ii**) Coating with parylene C (teal) creates a barrier that prevents absorption or leaching to and from the print. Reprinted by permission from *ACS Appl. Bio Mater.* 2023, 6, 8, 3079–3083. Copyright 2023 American Chemical Society

The Baker group successfully integrated a thin layer of parylene to insulate the cantilever [18]. Combining parylene with electrodeposited paint and mechanical abrasion to activate a small tip with parylene insulation achieved a high curvature radius, and the tip part remains active for further electrochemical measurements (Fig. 2). Additionally, focused ion beam (FIB) or cleanroom procedures are not required to fabricate the cantilever electrode. The parylene-insulated cantilever was characterized using cyclic voltammetry (CV) to record the current response of 15 mM Ru(NH_3_)_6_Cl_3_, revealing no oxidation or reduction of the electroactive species. Therefore, the thin-layer coating is sufficient to prevent charging current from flowing across the surface. The cantilever tip was then connected to a copper substrate while the current was applied to serve as a feedback loop for mechanical abrasion. The activated tip radius was about 200 nm or less and successfully induced redox signals. The coupled AFM tip can also be used to image the surface of the substrate. This work provides a direction for insulating multiple electrical instruments, such as probes, with parylene to prevent overcharging, facilitating further investigation in various aspects, including pH measurement [69–71].

### Aptasensing of gliadin with parylene-insulated screen-printed carbon electrodes

**Fig. 3:**
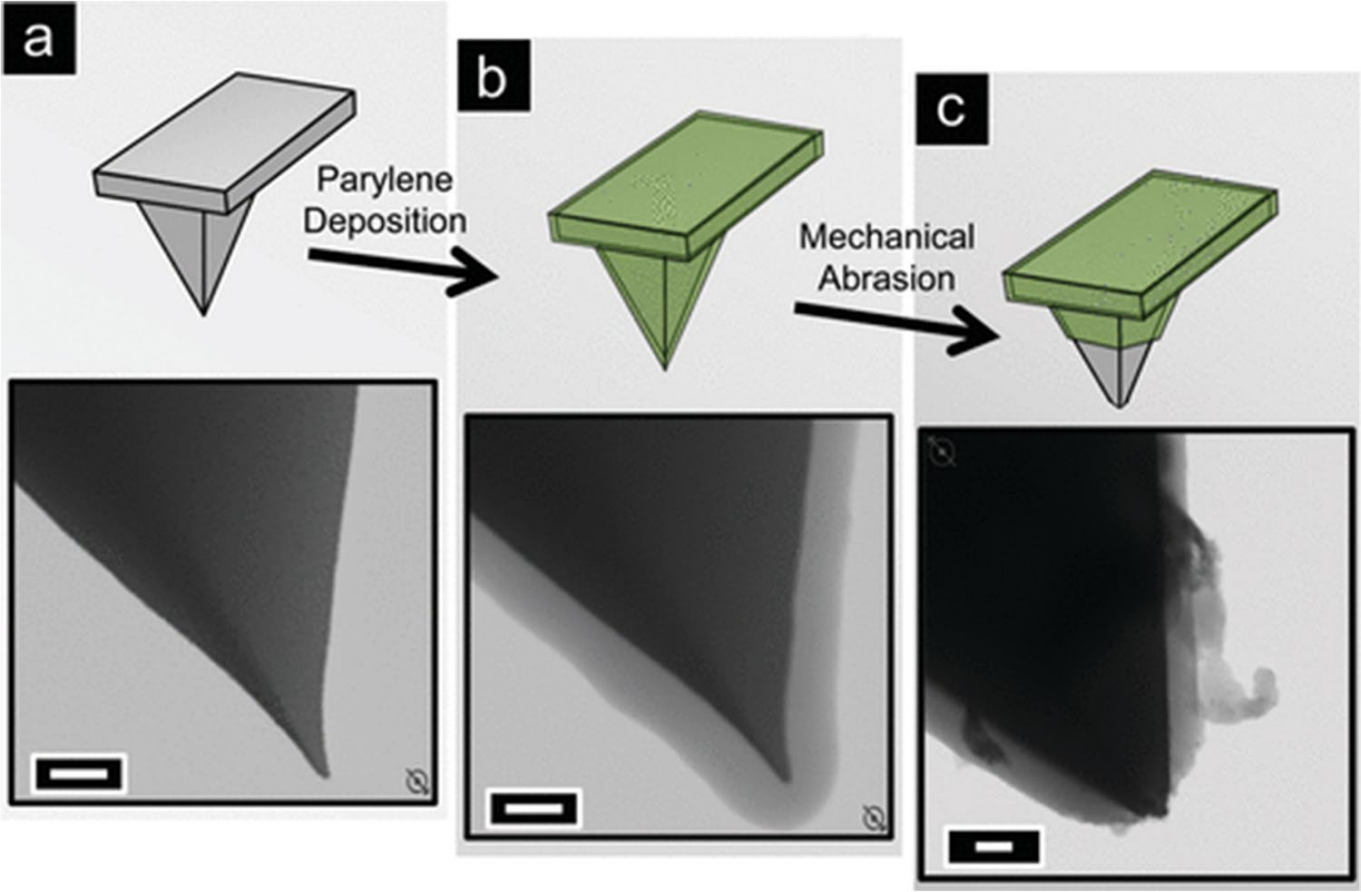
Scanning transmission electron microscopy (STEM) images of platinum-coated AFM tips. **a** Bare platinum tip directly from vendor, **b** parylene-coated tip, and **c** parylene-coated tip post exposure. Scale bar represents 200 nm. Reprinted from *Langmuir* 2011, 27, 22, 13925–13930. Copyright 2011 American Chemical Society

Parylene is also used as an insulator for screen-printed electrodes, making them biocompatible. One example is making a capacitive aptasensor for gliadin analysis (Fig. 3) [72]. Celiac patients, who suffer from an autoimmune disorder, require a gluten-free diet, and gliadin, a protein in gluten that can cause allergies, cannot be digested by intestinal enzymes and is therefore targeted for detection. Immunoassays, such as enzyme-linked immunosorbent assays (ELISA) and lateral flow devices (LFD), can have false negatives or nonquantitative results. Genomics-based and proteomics-based techniques are expensive and require specialized equipment and training. Aptamer-based biosensors (aptasensors) have garnered attention for immunoassays due to their cost-effectiveness, customizability, and the ability of selective aptamers to minimize cross-reactivity with similar molecules, thereby reducing the likelihood of false positives.

For capacitive aptasensing, a platform is needed that is well-insulated and has functional groups that can anchor the aptamers. The Hsieh group developed screen-printed electrodes (SPEs) with two parylene insulation layers for this purpose[72]. Parylene C, which is FDA-approved, is excellent in preventing water and gas penetration due to its high hydrophobicity and exceptional moisture impermeability. SPEs are coated with parylene C to ensure the electronic part is well-sealed. The coating of parylene C is covered by another layer of parylene AM (monoaminomethyl-substituted), and the amine groups of parylene AM are utilized as chemical anchors to achieve the immobilization of glutaraldehyde. Electrochemical assays were developed with the Gli4 aptamer bound to glutaraldehyde and then targeted to the immunotoxic 33-mer gliadin peptide.

### Application of parylene on multiplexing neurochemical detection

Carbon-fiber microelectrodes (CFMEs) with glass insulation are widely used for measuring neurotransmitters, to monitor neurochemicals with fast-scan cyclic voltammetry for high temporal resolution detection [73]. Recently, the Zestos group developed carbon-fiber multielectrode arrays (MEAs) insulated with parylene that enable multiplexed detection of neurochemicals, such as dopamine, serotonin, and adenosine [74]. Carbon fibers (CFs) inserted into the device are initially coated with parylene C to ensure the surface is insulated, guaranteeing rigid and stable detection. Then, a 532-nm green laser is used to partially expose the CF, creating an electroactive area. The electrochemical performance of MEAs is comparable to that of single-channel electrodes. Various waveforms can be applied to MEAs that contain parylene-coated CFs simultaneously to detect multiple neurotransmitters both in vitro and in vivo.

### Parylene improves hydrophobic properties in devices

Parylene is recognized for its outstanding hydrophobic properties, which prevent the intrusion of water and gases into electronics [10]. The range of water contact angles (WCAs) of parylene C is 85 to 97.2° [75]. However, a superhydrophobic surface, with a WCA of over 150°, can achieve self-cleaning and potentially control biofouling, and is expected to be present on parylene through surface modification. The biological response at the micro-device biointerface is influenced when surface wettability is changed [76]. One strategy to increase the hydrophobicity of parylene is plasma treatment, which renders the outer layer more hydrophobic without altering its overall properties. After a short-term treatment with oxygen plasma to introduce oxygen functional groups, the parylene surface is modified to become superhydrophilic, with a WCA of 0° [77]. Further treatment with a 1-min SF_6_plasma treatment changes the parylene surface from superhydrophilic to superhydrophobic, with a WCA reaching a maximum of 169.4 ± 0.3° [78]. These coating and treatment strategies can be used to enhance the hydrophobicity of devices, ensuring that water-based solvents do not adhere to the surface.

### Utilization of parylene on metal-organic frameworks for enhancing catalytic performance

Metal-organic frameworks (MOFs), a type of porous crystalline material, are synthesized using metal ions and clusters [79, 80]. MOFs exhibit high catalytic ability because of their customizable structure and extremely high surface area. However, MOFs need to be anchored to a substrate, often to help them maintain their structure and catalytic properties [81, 82]. Several methods have been developed to prevent the MOF structure from degrading, including the use of polydimethylsiloxane (PDMS) coating [83]. However, the preparation process for this method is complex and challenging, and the PDMS surface cannot be guaranteed to be smooth. Chemical vapor deposition of parylene on the surface of MOFs is considered a fast and efficient approach for coating MOFs, maintaining a hydrophobic surface, and ensuring the entire structure remains stable and intact, which makes parylene a better choice than PDMS [10, 15]. The Chen group fabricated MOFs using UiO-66-NH_2_, ZIF-8, and HKUST-1, and then coated them with parylene N (PN) to assess the stability of surface-modified MOFs [84]. Before and after parylene coating on the MOFs, XRD data showed no change in the intrinsic structures of all materials. After stirring PN-coated UiO-66-NH_2_, ZIF-8, and HKUST-1, respectively, in NaOH, HCl, and water, XRD data showed no change in the intrinsic structures of the materials and no significant change in Brunauer-Emmett-Teller (BET) specific surface area. Therefore, PN helps stabilize the fabricated MOFs’ structure and resist the influences of water, acid, and base. To further test the catalytic activity and recyclability of coated samples, PN-coated HKUST-1 was utilized in the Knoevenagel condensation reaction, yielding 93.3% and retaining 92% of its catalytic activity after three cycles. Table 1 summarizes applications of parylene as an insulator in various analytical techniques, leveraging its biocompatibility, insulation properties, and flexible modified surface.

**Table 1:** Summary of insulative parylene utilized for various techniques

Parylene type	Thickness (nm)	Application	Note	Ref
C	N/A	Protect cultured cells from toxic 3D-printed structure	Biocompatibility	42
C	700	Lower charging current of the cantilever for SECM-AFM	Insulation	18
C AM	1000 400	Electronic sealing (C) Surface modification (AM)	Insulation and surface modification	72
C	N/A	Provide rigidity and stability for multiplexed carbon fibers	Biocompatibility and insulation	74
C	N/A	Improve hydrophobicity with oxygen and SF_6_ plasma	Hydrophobic modification	78
N	N/A	Modified MOF surface to retain catalytical ability and recyclability	Insulation	80

## Applications of pyrolyzed parylene as graphene-based sensors

### Pyrolyzed parylene C electrodes for cyclic voltammetry

**Fig. 4:**
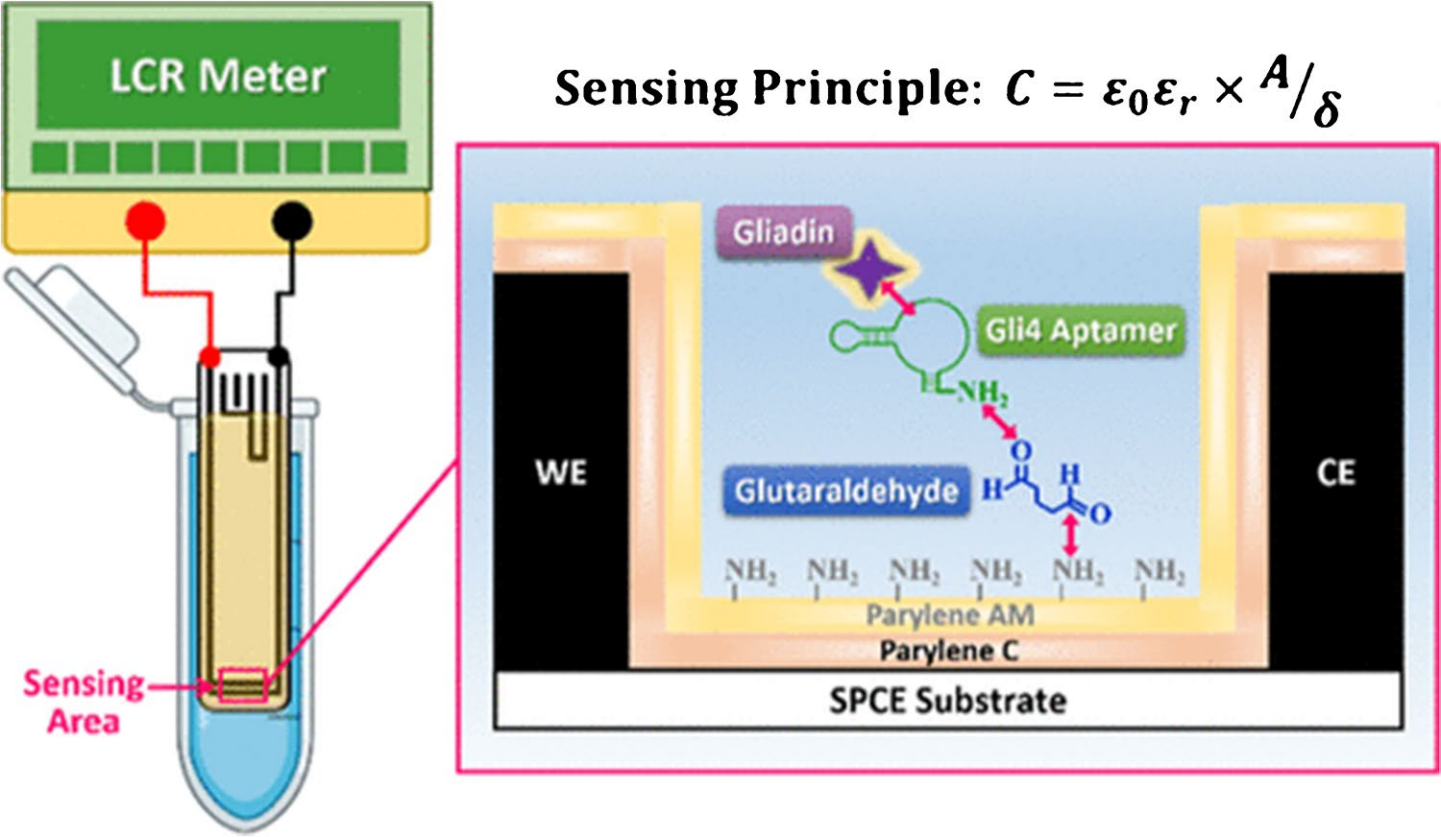
Schematic illustration of the capacitive aptasensor for gliadin analysis. Parylene C and parylene AM were sequentially coated onto an SPCE via CVD, followed by immobilization of the 5′-NH_2_-modified Gli4 aptamer onto the sensing area using glutaraldehyde. The prepared SPCE was connected to the LCR meter probes and immersed in the testing solution. The change in capacitance resulting from the affinity binding between the aptamer and gliadin was monitored and calculated for quantification. Reprinted from *ACS Sens.* 2024, 9, 7, 3689–3696. Copyright 2024 American Chemical Society

While parylene is an excellent material for insulating devices [8, 85] and is biocompatible [10, 42], it can also be pyrolyzed into carbon and then used as an electrochemical sensor. The Baker group synthesized pyrolyzed parylene C (PPC) by using high temperatures to induce the pyrolysis of parylene (Fig. 4). Parylene possesses an extremely high resistance, and no current flows inside the structure [19]. Parylene is pyrolyzed at 900 °C under a N_2_atmosphere, as the benzene rings undergo structural reformation to form a conductive graphitic material consisting of multiple graphene layers. PPC was characterized by Raman spectroscopy, and the D and G bands, which originate from the edge and basal planes of graphite, are present in the spectra [86, 87]. Before pyrolysis, the parylene surface is smooth and defect-free. However, defect sites, which favor the adsorption of neurotransmitters, were generated on the sample surface during the high-temperature pyrolysis. Cyclic voltammetry (CV) was used to detect Ru(NH_3_)_6_Cl_3_and dopamine at PPC electrodes. Thus, PPC possesses good electrical conductivity and can be applied for the electrochemical characterization of neurotransmitters. Pyrolyzed parylene is also used for the fabrication of AFM probes, nanoporous membrane arrays, and electrochemical immunoassays [20, 88, 89].

### Pyrolyzed parylene N electrodes with fast-scan cyclic voltammetry

To enable the application of pyrolyzed parylene electrodes for real-time tracking of neurotransmitters, microelectrodes are needed that can be coupled with fast-scan cyclic voltammetry (FSCV). FSCV is a technique that uses a high scan rate (400 V/s) triangular waveform, which is approximately 1000 times higher than a typical cyclic voltammetry scan rate. Thus, small electrodes are needed that have stable background currents that can be subtracted out. Compared to traditional CV, FSCV offers a high temporal resolution, enabling real-time recording.

**Fig. 5:**
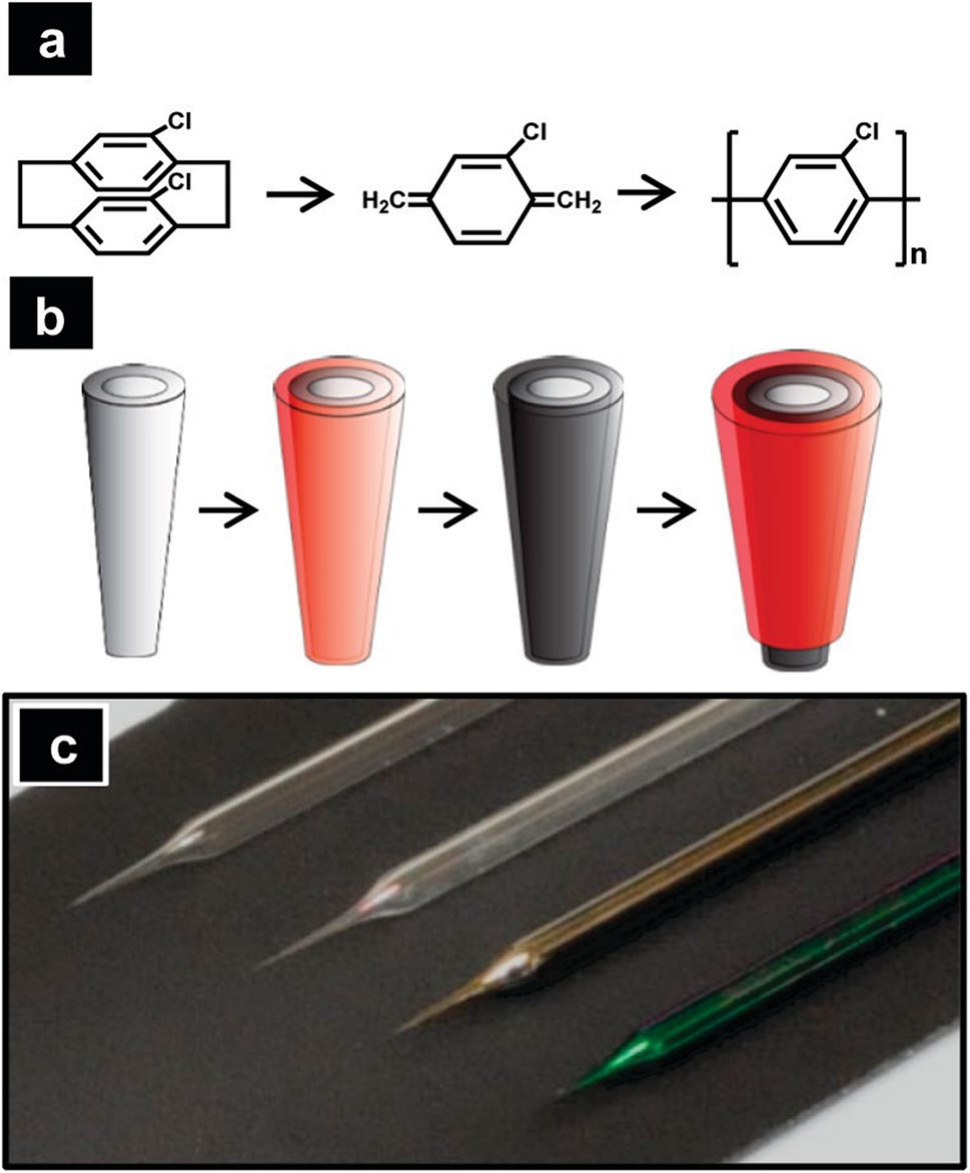
**a** Parylene reaction scheme. **b** A schematic of the microelectrode fabrication process from a bare pipet to a final pyrolyzed microelectrode. A bare quartz pipet (far left) is coated with a thin layer of parylene C and subsequently pyrolyzed under an inert atmosphere. The pipet is then masked and insulated with another layer of parylene C to form a carbon electrode (far right). **c** Optical micrographs of pipets at different stages of this process. From left to right: a bare pipet, a pipet after initial parylene C deposition, after pyrolysis of parylene C, and a final parylene-insulated carbonaceous pipet. Reprinted from *Anal. Chem.* 2011, 83, 13, 5447–5452. Copyright 2011 American Chemical Society

**Fig. 6:**
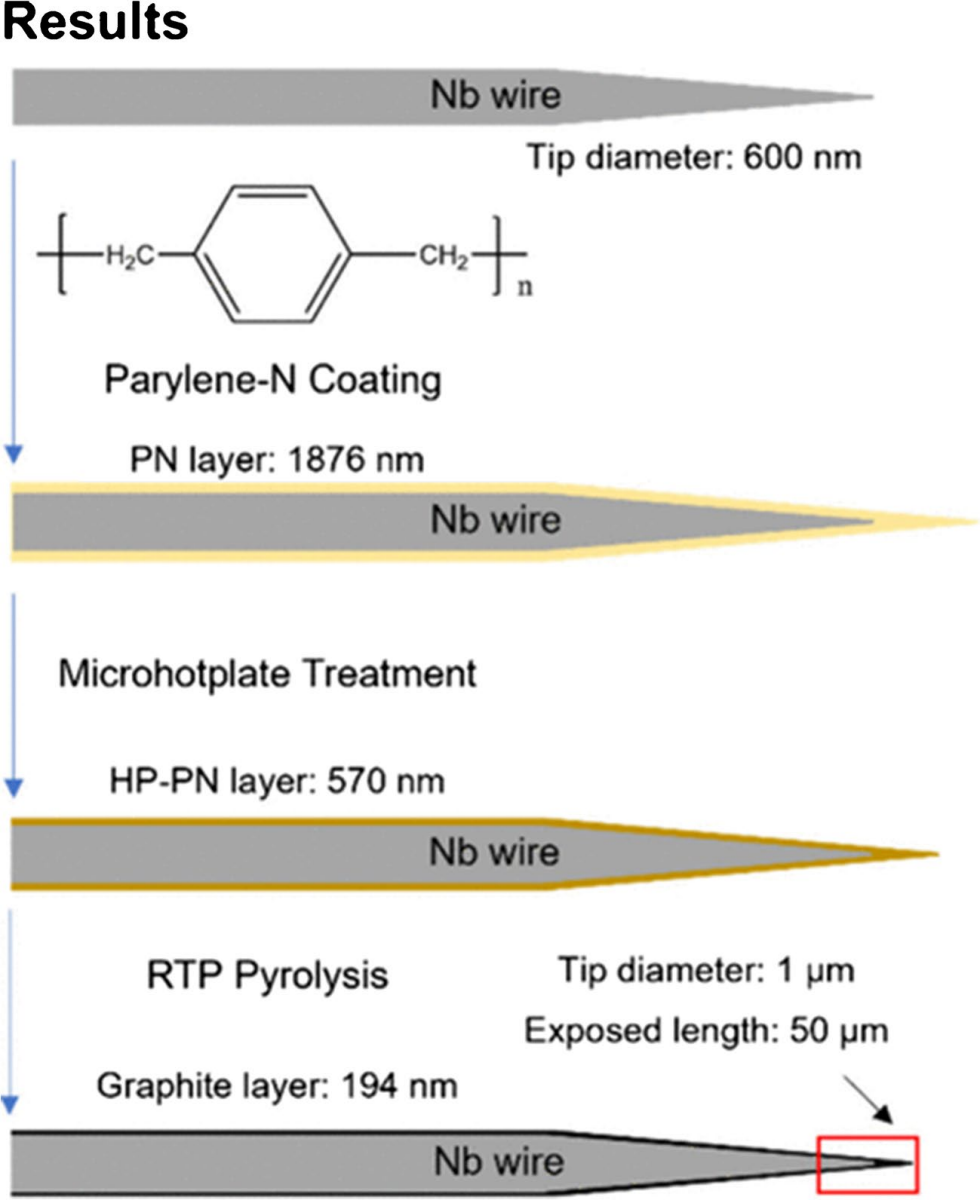
Overview of pyrolyzed parylene electrode fabrication. First, parylene N is deposited on a wire; then, parylene is treated on a microhotplate, and finally, pyrolysis is performed in a rapid thermal processor. The result is a thin film of graphite on a Nb wire. Thicknesses are given for 6 g of the precursor. Reprinted from *ACS Electrochem*. 2025, 1, 5, 730–740. Copyright 2025 American Chemical Society

The Venton group generated microelectrodes by pyrolyzing parylene N (PN) deposited on niobium (Nb) wires (Figs. 5 and 6) [21]. Different amounts of dimer powder, 1 g, 6 g, and 12 g, were initially loaded for parylene deposition. Parylene-coated Nb wires were pre-annealed using a microhotplate ramp to 350 °C, which caused the parylene to undergo volumetric loss and the addition of oxygen functional groups, but not to graphitize. Therefore, a rapid thermal processor was used to induce pyrolysis of parylene by applying two conditions: (1) 600 °C at 9 Torr for 10 min under argon, and (2) 950 °C at 1 Torr for 10 min under argon. These recipes are commonly used for carbonizing 3D-printed polymer structures [90–92]. The results from the three different thicknesses of the precursor were thin films of PN samples, ranging from 81 to 476 nm. Parylene and hot-plate-treated parylene are highly resistant to electrical flow, but RTP-PN is conductive, and the measured resistance of RTP-PN (6 g) is 320 Ω. The estimated conductivity obtained from the COMSOL simulation is approximately 3000 S/m, which falls within the range of the conductivity of glassy carbon.

RTP-PN (6 g) microelectrodes were used for neurochemical recording with FSCV, targeting the analytes dopamine (DA), adenosine (AD), and serotonin. Compared to carbon-fiber microelectrodes (CFMEs), the common electrode used for FSCV, pyrolyzed parylene N modified microelectrodes (PPNMEs) had higher sensitivities for all tested neurochemicals. PPNMEs had higher oxygen functional groups and richer defect sites than CF, as confirmed by XPS and Raman spectra, which make them highly sensitive for neurotransmitter detection. Additionally, PPNMEs resist fouling, which originates from polymerized serotonin molecules during electrooxidation or from biofouling caused by the attachment of brain tissue and unfolded proteins. Therefore, PPNMEs are appropriate for in vivo testing and recording neurochemical signals in vivo.

### Laser-induced graphene from parylene C applied for microsupercapacitor

While pyrolyzed parylene has been produced using rapid thermal processing [19, 21], the pyrolysis procedures for parylene require two steps and special equipment. A CO_2_ infrared laser, used for graphitizing commercially available polyimide (PI), can serve as an alternative for treating parylene through the photothermal conversion of sp^3^ carbon to sp^2^carbon [22]. LIG can be applied to sensors, such as strain sensors, as well as the fabrication of microsupercapacitors (MSCs) [24, 93, 94]. Carbonization of parylene with a CO_2_infrared laser enables devices to be coated with ultrathin membranes [95]. LIG from parylene was then applied to the fabrication of MSCs to replace lithium-ion batteries (LIBs). LIBs have some limitations, including a decrease in energy per volume on the microscale, the use of hazardous electrolytes, and impracticality for wearable sensors [96]. The porous structure of LIG enables the MSC to have a high conductivity and a large surface area [23]. Therefore, MSCs possess superior power density compared to LIBs while ensuring a safe application without the use of hazardous electrolytes. MSCs can achieve a faster charge/discharge process and endure more cycles than LIBs [97, 98]. LIG from parylene can be used for MSCs production, and the capacity is 1.66 mF/cm^2^, 96% cyclic stability (0.5 mA/cm^2^), and an energy density of 0.19 μWh/cm^2^.

### Plasma deposited and pyrolyzed parylene C for the fabrication of electrochemical immunoassays

**Fig. 7:**
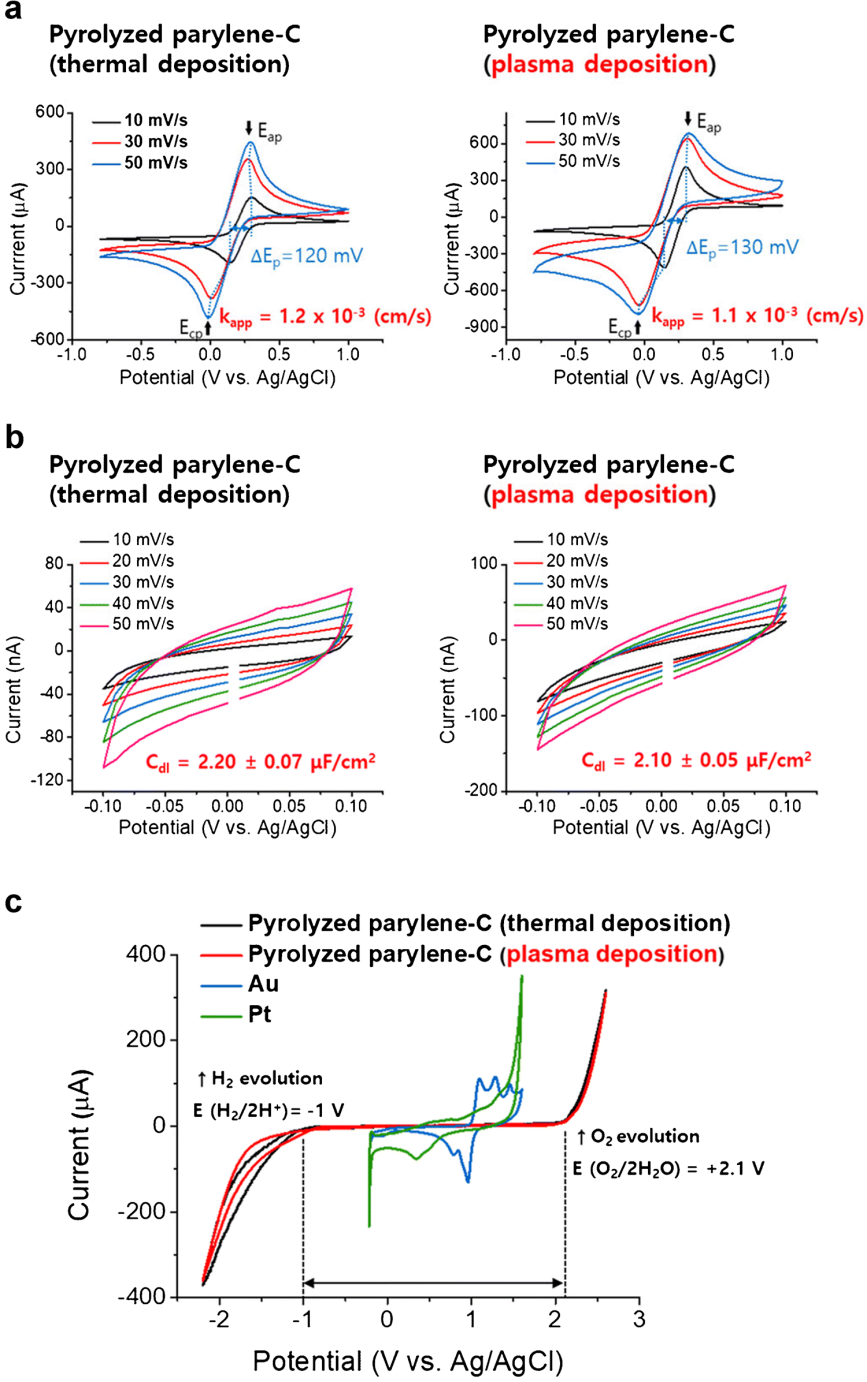
Electrochemical properties of pyrolyzed parylene C obtained via thermal and plasma deposition. **a** Apparent electron transfer rate (*k*_app_) measured using ferricyanide, **b** double-layer capacitance (*C*_dl_), and **c** electrochemical window. *E*_ap_, anodic peak potential; *E*_cp_, cathodic peak potential; Δ*E*_p_, peak potential separation. Reprinted from *Analyst*, 2022, 147, 3783–3794. Copyright 2022 Royal Society of Chemistry

The Pyun group pyrolyzed both thermally and plasma-deposited parylene and then applied them for electrochemical immunoassay fabrication. After high-temperature pyrolysis, all functional groups of both types of parylene were removed, and the D and G peaks were present, indicating the formation of graphene layers and defect sites [99] with highly disordered structures [100, 101]. XPS spectra revealed that C, O, and Cl are present in parylene deposited by both methods, whereas only N is incorporated into the parylene structure during plasma deposition. Figure 7 shows that the electrochemical properties are similar for the two types of deposition. The apparent electron transfer rates (Fig. 7a) are similar to other carbon electrodes, the double-layer capacitance is lower than metals and similar to other carbon electrodes (Fig. 7b), and the electrochemical window was larger than conventional graphite (Fig. 7c). Therefore, there are slight differences in the structure of parylene deposited by both methods; however, they become similar after pyrolysis. The advantage of the plasma-assisted method is that it deposits parylene at a faster rate.

Human hepatitis C was diagnosed using a pyrolyzed carbon electrode coated with parylene C, deposited on the substrate via conventional thermal deposition and plasma deposition. Initially, the hepatitis C virus (HCV) antigen was deposited on a substrate surface. Anti-HCV antibody and secondary antibody, both labeled with horseradish peroxidase (HRP), were bound to the HCV antigen. 3,3′,5,5′-tetramethylbenzidine (TMB) binds to the antigen and is oxidized to first (ox-TMB1) and secondary products (ox-TMB2) in the presence of HRP and H_2_O_2 _[102, 103]. The ox-TMB2 is detected using pyrolyzed parylene C electrodes with cyclic voltammetry (CV) scan from 0.3 to 0.7 V. They compared pyrolyzed parylene C deposited via two deposition methods, and pyrolyzed parylene electrodes showed a similar electrochemical performance in the analysis of ox-TMB2. Chronoamperometry, square wave voltammetry (SWV), and differential pulse voltammetry (DPV) can also be applied in electrochemical immunoassays and the analysis of hepatitis C.

Plasma-assisted deposition of other parylene derivatives, such as parylene AM and H, is further utilized to create surface functional groups that facilitate protein immobilization for immunoassay fabrication [104]. An amine group is attached to the benzene ring in parylene AM, while a formyl group is in parylene H. The advantage of the plasma deposition method is that all functional groups remain on the surface, and the cross-linked form is more hydrophilic [34]. Plasma-deposited parylene films were used for the detection of SARS-CoV-2 when immobilizing antigens for immunoassays [33]. Due to crosslinking during plasma deposition, parylene films possess groups that are not initially present in either dimer or thermally deposited parylene, including C=O stretching and C-O stretching for parylene AM and N-H bending and C-O stretching for parylene H [33]. Parylene H is used for the direct immobilization of HRP and green fluorescent protein (GFP). Plasma-deposited parylene AM and H, respectively, increased the immobilization efficiencies of HRP by 51.8% and 58.7% and GFP by 30.2% and 267.0%. The sensitivities of the SARS-CoV-2 receptor-binding domain (RBD) increased, and the limit of detection (LOD) was lowered to 0.03 nM and 0.08 nM for plasma-deposited parylene AM and H, respectively. In comparison, the LODs for thermally deposited parylene are 0.90 nM and 2.64 nM, respectively. Plasma-deposited parylene films facilitate covalent bonding between antibodies and functional groups, including amines and formyl groups. Immunoassays developed using plasma-deposited parylene AM and H present a promising method for detecting SARS-CoV-2 and have the potential to be applied to the detection of other viruses. Table 2 summarizes pyrolyzed parylene obtained by different methods and their analytical applications in CV, FSCV, microsupercapacitors, and immunosensors.

**Table 2:** Summary of pyrolyzed parylene utilized for various techniques

Parylene type	Source	Mass (g)	Pyrolyzed thickness (nm)	Application	Note	Ref
C	High-temp under vacuum	N/A	N/A	Microelectrode: dopamine and Ru(NH_3_)_6_Cl_3_ with CV	N/A	19
N	RTP	1	81 ± 10	Microelectrode: thinner coating	N/A	21
N	RTP	6	194 ± 20	Microelectrode: optimal thickness, dopamine and serotonin with FSCV	3000 S/m (conductivity)	21
N	RTP	12	476 ± 35	Microelectrode: thicker coating	N/A	21
C	CO_2_ laser (N_2_)	N/A	27	Microsupercapacitor fabrication: thinner coating	178.5 Ω/sq (Resistivity)	95
C	CO_2_ laser (Air)	N/A	46	Microsupercapacitor fabrication: thicker coating	9.4 Ω/sq (Resistivity)	95
C	High-temp/vacuum	N/A	21	Immunosensor for human hepatitis C: thinner coating	N/A	20
C	High-temp/vacuum	N/A	167	Immunosensor for human hepatitis C: thicker coating	N/A	20

## Conclusions

Parylene is a promising material that can be used to insulate various devices due to its excellent gas and moisture impermeability, as well as its biocompatibility. It is commonly chemically vapor-deposited using high temperatures, but an alternative deposition method is plasma deposition, which results in a cross-linked structure distinct from the conventional linear chain structure. Parylene coating benefits cell culture devices by covering resins from 3D printing, reducing leachate contaminants over time, thus facilitating long-term monitoring of cells and tissues. Parylene is also helpful in insulating SECM and AFM tips to prevent overcharging. Parylene can be used to improve the thermal insulating performance of SiO_2_ aerogel. Both parylene C and parylene N are pyrolyzed at high temperatures, and the pyrolyzed parylene sensors are used for fabricating microelectrodes for neurochemical detection or immunoassays for detecting gluten or viruses. Pyrolyzed parylene N exhibits higher electrochemical sensitivities than CFMEs and possesses anti-fouling and biofouling properties, making it suitable for in vivo recording applications. The crosslinked structure achieved by plasma-assisted treatment facilitates the immobilization efficiencies for immunoassays. Thus, parylene serves as a good insulator, and pyrolyzed parylene serves as a good conductive sensor for many analytical sensors and devices.

Parylene is likely to grow in its applications for analytical devices and sensors. For example, as 3D printing grows for analytical devices, there may be other applications in addition to cell culture, such as gas sensing or environmental analysis, where coating with parylene will protect the contents of the device from leachates. Implantable devices will continue to be made with parylene, as parylene is biocompatible, but the future will be in integrated devices where sensors can be selectively pyrolyzed on the surface. For example, CO_2_ infrared laser or other lasers could be used to selectively pyrolyze parylene as to LIG. This approach can be applied to the fabrication of microsupercapacitors and array-based sensors. Finally, LIG from parylene or high-temperature-pyrolyzed parylene could be applied to the development of multichannel arrays for neurotransmitter recording. Thus, the application of parylene to fabricated integrated analytical devices and sensors is likely to increase.
